# Multifunctional Meta-Devices for Full-Polarization Rotation and Focusing in the Near-Infrared

**DOI:** 10.3390/mi15060710

**Published:** 2024-05-28

**Authors:** Hengyi Wan, Kai Ou, Hui Yang, Zeyong Wei

**Affiliations:** 1 Institute of Precision Optical Engineering, School of Physics Science and Engineering, Tongji University, Shanghai 200092, China; 2130945@tongji.edu.cn; 2 MOE Key Laboratory of Advanced Micro-Structured Materials, Shanghai 200092, China; 3 Shanghai Frontiers Science Center of Digital Optics, Shanghai 200092, China; 4 School of Physics and Electronics, Hunan Normal University, Changsha 410081, China; yangh1023@126.com

**Keywords:** full-polarization generation, polarization rotation, focusing, all-dielectric metasurfaces

## Abstract

The creation of multi-channel focused beams with arbitrary polarization states and their corresponding optical torques finds effective applications in the field of optical manipulation at the micro-nanoscale. The existing metasurface-based technologies for polarization rotation have made some progress, but they have been limited to single functions and have not yet achieved the generation of full polarization. In this work, we propose a multi-channel and spatial-multiplexing interference strategy for the generation of multi-channel focusing beams with arbitrary polarization rotation based on all-dielectric birefringent metasurfaces via simultaneously regulating the propagation phase and the geometric phase and independently controlling the wavefronts at different circular polarizations. For the proof of concept, we demonstrate highly efficient multi-channel polarization rotation meta-devices. The meta-devices demonstrate ultra-high polarization extinction ratios and high focusing efficiencies at each polarization channel. Our work provides a compact and versatile wavefront-shaping methodology for full-polarization control, paving a new path for planar multifunctional meta-optical devices in optical manipulation at micro–nano dimensions.

## 1. Introduction

The optical polarization manipulation at the micro-nano scale has important applications for optical tweezers [1], optical trapping [2], and particle sorting [3] in response to the demand for modern optical micro-manipulation [4,5]. The initial endeavors for the alignment and rotation of individual nanoscale plasmonic structures are achieved via the optical torque generated by rotating the linear states of polarization (SOPs) of the laser beam. The research has introduced a special and important modality of optical control of nanoplasmonic structures [6]. Typically, to achieve the non-contact optical polarization techniques for manipulating the orientations of nanoparticles and nanoaggregates, the desired SOPs generally can be obtained by rotating the linearly polarized incidence [7]. This results in the inevitable use of heavily cascaded mechanical configurations in traditional optical systems, which severely restrict their on-chip integration and miniaturization [8].

Optical metasurfaces represent two-dimensional artificial structured patterns comprising subwavelength meta-units with planar and easy integration nature [9,10,11,12,13,14,15,16,17,18]. These meta-structures proficiently and precisely manipulate multidimensional parameters of electromagnetic waves, including phase, amplitude, and polarization, enabling the tailored shaping of light waves at the subwavelength scale [19,20,21,22,23,24,25,26,27,28]. The multifunctional and integrated characteristics of meta-devices [29,30] bring unprecedented convenience to the development of ultra-compact optical systems, such as microfluidic chips [31], metasurface particle sorting [32], etc. Remarkable polarization-controlled meta-devices can customize the polarization state of light to generate optical torque to capture and manipulate particles [33,34]. However, it is necessary to control the polarization angle of the incident light to achieve the corresponding output SOPs [35,36]. Additionally, researchers have yet to achieve arbitrary SOPs rotation via a single metasurface due to the lack of a general design approach for multiplexing the phase and polarization distributions [35,37].

In this paper, we proposed a multi-channel interference and spatial-multiplexing methodology to achieve arbitrary SOPs rotation based on all-dielectric meta-devices with multi-focus. Benefiting from simultaneously controlling the geometric phase and the transmission phase, the interference superposition principle introduces the multi-channel full-polarization vector control. For the proof of concept, we demonstrate a high-efficiency multi-channel meta-device with near-continuous linear polarization rotation and focusing at the near-infrared wavelength of λ=1.55 μm. The maximum extinction ratio of the focal spot’s intensity profile reaches over 300, and the extinction ratio of each channel is up to 100, which verifies the designed SOPs of the focal spots. Furthermore, we have developed a six-focus and full-polarization-controlled metalens, thereby affirming the viability of arbitrary polarization rotation. Our primary focus lies in the development of non-contact technologies for achieving the controlled manipulation, orientation, and rotation of nanoparticles and nanoaggregates. This is facilitated through polarization rotation enabled by polarization-multiplexed metasurfaces, carrying implications for future optical assembly and micromachined components crucial in driving nanomachine applications.

## 2. Results and Discussion

### 2.1. Principle of Multi-Channel Polarization Rotation and Focusing Based on Metasurfaces

**Figure 1:**
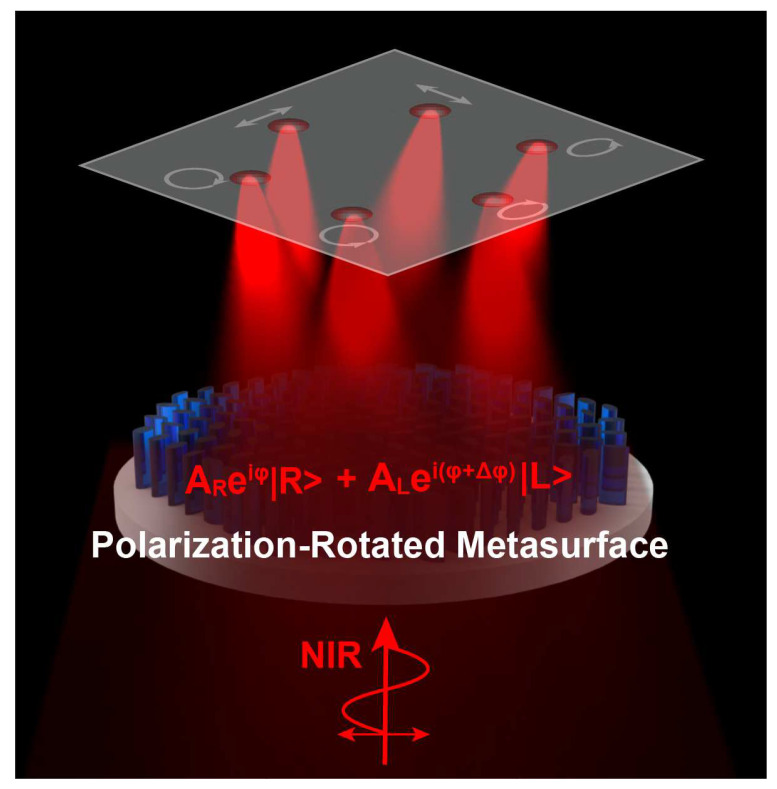
Schematic illustration of high-efficiency multi-channel rotated polarization focusing meta-device. The all-dielectric polarization-rotated metasurface operates in transmission mode. Near-infrared beams with XLP incident on the meta-device which creates multi-channel rotated polarization intensity distribution.

Figure 1 schematically illustrates the high-efficiency focusing with a full-polarization state via all all-dielectric metasurface with the diameter of D=40 μm. It is capable of efficiently transforming normal incidence into a multi-channel focused beam bearing different SOPs at a near-infrared (NIR) wavelength of λ=1.55 μm. Each channel can be given arbitrarily superimposed circular polarizations (CPs) components by combining both the propagation and geometric phases, which introduce full-polarization multi-channel wavefront shaping. For a normal incident optical field $E_{in}$ with an arbitrary state of polarization (SOP), it can be decomposed into the superposition of two CP components:

(1) $$E_{in}=a_{R}e^{i\varphi_{R}}|R>+a_{L}e^{i\varphi_{L}}|L>$$

where $a_{R}$, $a_{L}$, $\varphi_{R}$, and $\varphi_{L}$ are the amplitude and phase for the right-handed circular polarization (RCP, $|R>={\left[1,-i\right]}^{T}$) and left-handed circular polarization (LCP, $|L>={\left[1,i\right]}^{T}$) components, respectively. For the *x*-linearly polarized (XLP) incident light, $a_{R}=a_{L}=\sqrt{2}/2$, $\varphi_{R}=\varphi_{L}=0$. Here, for the design of the multifunctional polarization-rotated and focusing meta-devices, the target SOP of the output light $E_{out}$ modulated by the metasurfaces can be expressed as

(2) $$E_{out}=A_{R}e^{i\Phi_{R}}|R>+A_{L}e^{i\Phi_{L}}|L>$$

where $A_{R}$ and $A_{L}$ indicate the number of RCP and LCP components, while $\Phi_{R}$ and $\Phi_{L}$ are the synthetic phases of the multiple-beam interference, respectively. Therefore, the output wavefront manipulation of an arbitrary SOP can be achieved by controlling the values of $A_{R}$, $A_{L}$, $\Phi_{R}$, and $\Phi_{L}$:

(3) $$\Phi_{R}=arg\left(\underset{j}{\overset{n}{\sum }}A_{j}^{R}e^{i\varphi_{j}^{R}}\right)$$

(4) $$\Phi_{L}=arg\left(\underset{j}{\overset{n}{\sum }}A_{j}^{L}e^{i(\varphi_{j}^{L}+\Delta \varphi )}\right)$$

where $A_{j}^{R}$, $A_{j}^{L}$, $\varphi_{j}^{R}$, and $\varphi_{j}^{L}$ are the amplitude and phase of the right-handed and left-handed circular polarization components at each position. *j* is the $j^{th}$ targeted light beam and *n* is the total number of targeted beams. Δφ∈[0,2π], is the elaborately endowed phase shift on the output LCP component. Here, to achieve equal energy in every channel, the normalized electric field intensity $A_{j}^{R}=A_{j}^{L}=\sqrt{1/n}$ is assumed. The polarization state of each channel is superimposed by the CP components, and the interference synthetic phases of left-hand and right-hand circularly polarized light can be designed independently. The desired phase shifts $\varphi_{j}^{R}$ and $\varphi_{j}^{L}$ for the orthogonal circular polarizations should be functions of the spatial coordinates (x,y):

(5) $$\varphi_{j}\left(x,y,0\right)=-\frac{2\pi }{\lambda }\left(\sqrt{({x-x_{j})}^{2}+({y-y_{j})}^{2}+f^{2}}-f_{j}\right)$$

where $f_{j}=\sqrt{{\left(x_{j}\right)}^{2}+{\left(y_{j}\right)}^{2}+f^{2}}$ and $\left(x_{j},y_{j}\right)$ are the focal length and Cartesian coordinate of the focus at the predesigned focal plane, respectively. f=40 μm is the distance from the metasurface to the focal plane. The output polarization state for each position can be derived through the superposition of orthogonal circular polarizations, as delineated by Equation (2). According to the predefined focal positions, the interference phase is obtained using Equations (3)–(5). Next, a phase map accomplished via selecting the appropriate meta-atoms for each pixel coordinate (x,y) on the metasurface. The specific details are discussed below.

The birefringent metasurfaces composed of elliptical cylindrical meta-atoms can be described by the following Jones matrix:

(6) $$J\left(x,y\right)=R(-\theta (x,y))\left[\begin{array}{cc} e^{i\varphi_{x}(x,y)} & 0\\ 0 & e^{i\varphi_{y}(x,y)} \end{array}\right]R(\theta (x,y))$$

where $\varphi_{x}(x,y)$ and $\varphi_{y}(x,y)$ represent the spatial propagation phase profiles imparted by the meta-atoms under linearly polarized incidence along the two symmetry axes at each coordinate (x,y). θ(x,y) represents the orientation angle of the meta-atom which determines the geometric phase shift, and *R* is a 2 × 2 rotation matrix. The polarization control of incident light by the polarization control metasurface can be described by $J\left(x,y\right)\left|L>=e^{i\Phi_{R}\left(x,y\right)}\right|R>$ and $J\left(x,y\right)\left|R>=e^{i\Phi_{L}\left(x,y\right)}\right|L>$. After performing the matrix inversion of these two equations, we can calculate the required Jones matrix as

(7) $$J\left(x,y\right)=\left[\begin{array}{cc} \frac{e^{i\Phi_{R}\left(x,y\right)}+e^{i\Phi_{L}\left(x,y\right)}}{2} & \frac{{ie}^{i\Phi_{L}\left(x,y\right)}-{ie}^{i\Phi_{R}\left(x,y\right)}}{2}\\ \frac{{ie}^{i\Phi_{L}\left(x,y\right)}-{ie}^{i\Phi_{R}\left(x,y\right)}}{2} & \frac{{-e}^{i\Phi_{R}\left(x,y\right)}-e^{i\Phi_{L}\left(x,y\right)}}{2} \end{array}\right]$$

According to Equations (6) and (7), we can obtain the corresponding relationship between the target required phase spectrum ($\Phi_{R}$ and $\Phi_{L}$) and the spatial phase distribution ($\varphi_{x}$ and $\varphi_{y}$) introduced by the meta-atoms:

(8) $$\Phi_{R}\left(x,y\right)=\varphi_{x}\left(x,y\right)-2\theta (x,y)$$

(9) $$\Phi_{L}\left(x,y\right)=\varphi_{y}\left(x,y\right)+2\theta \left(x,y\right)-\pi$$

(10) $$\theta \left(x,y\right)=\left[\Phi_{R}\left(x,y\right)-\Phi_{L}\left(x,y\right)\right]/4$$

Equations (8)–(10) demonstrate that the elaborate design of the phase compensation and rotation angle of the meta-atoms allows for precise decoupling control of the target circularly polarized light (CP). That is, the phase shift provided by the meta-device can match the required interference synthetic phase, as expressed by Equations (3)–(5). θ represents the orientational angle of the selected meta-atoms. Specifically, polarization-controlled metasurfaces can be achieved by adjusting the structural parameters of the meta-atoms.

### 2.2. Multi-Channel Linear Polarization Rotation and Focusing

**Figure 2:**
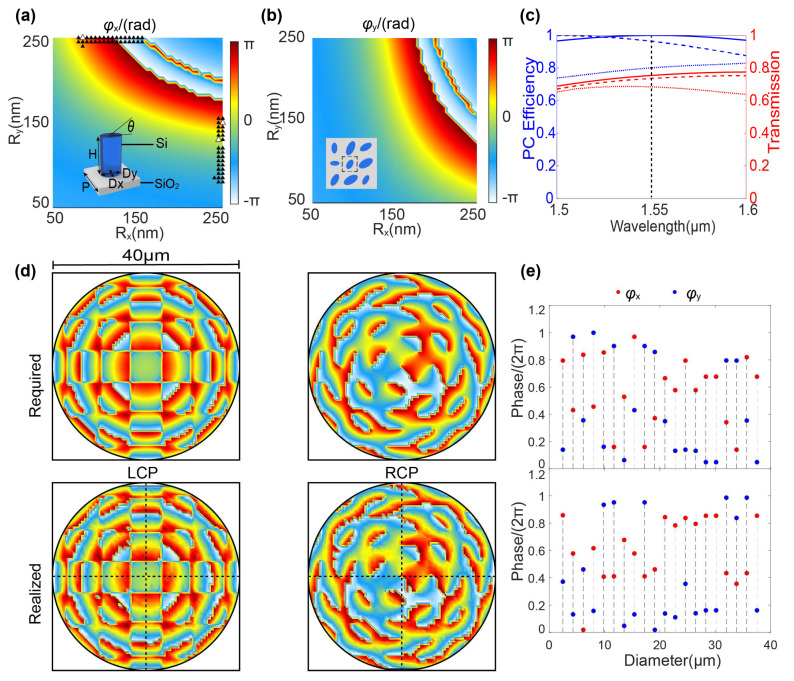
An efficient and high-extinction ratio multi-channel polarization rotation design method based on all-dielectric metasurfaces. (**a**,**b**) Phase maps of elliptical nanopillar as a function of the major-axis *Rx* and minor-axis *Ry* for *x*- and *y*-polarizations, respectively. Triangular markers denote the specific meta-atoms selected during the design process. The inset in (**a**) annotates the structural parameters of the meta-atom and the inset in (**b**) illustrates the cell lattice of meta-atoms. (**c**) The polarization conversion efficiency (PCE) and transmittance of the meta-atoms marked by the white triangle at a design wavelength of 1.55 μm in (**a**). The polarization conversion efficiency (PCE) is calculated as the ratio of transmitted optical power $\left(E_{CP}\right)$ with opposite helicity to the total power ($E_{total}$):$PCE=E_{CP}/E_{total}$. (**d**) The realized and required phase profiles for multi-channel superposition. (**e**) Phase shifts for *x*- and *y*-polarized incidences for the selected and optimal meta-atoms used in the design.

**Figure 3:**
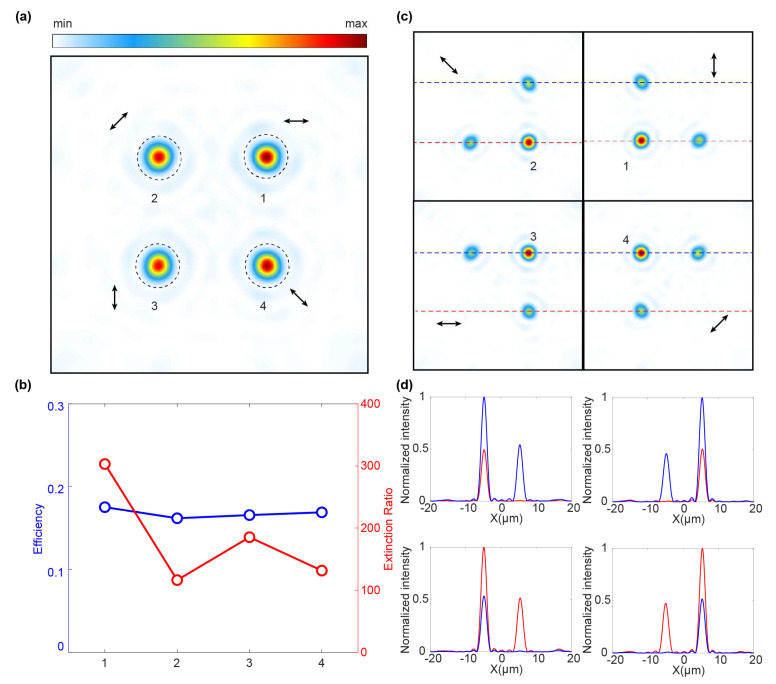
Simulation characterization of linearly polarization rotation metalens. (**a**) Focal plane intensity distribution with multi-channel focused polarization rotation. Focal spots numbered 1–4 correspond to uniform polarization rotations of 0–180°, respectively. (**b**) Calculated efficiency and polarization extinction ratio for each focal spot. (**c**) The intensity distribution of each focus is analyzed using orthogonal linear deviation states. (**d**) The normalized intensity distribution at the dotted line position in (**c**) after analyzing the intensity distribution of each focusing.

Figure 2a,b show the simulated phase shifts $\varphi_{x}$ and $\varphi_{y}$ of the silicon elliptical meta-atoms as a function of the semi-major axis (*Rx* varies from 50 to 250 nm) and semi-minor axis (*Ry* varies from 50 to 250 nm) under *x*- and *y*-polarized incidence with a wavelength of λ=1.55 μm, respectively. The cell lattice of the meta-atoms is P=0.615 μm, and the height of the nanopillars is H=1 μm as shown in the inset annotation in Figure 2a. To validate the design methodology, we initially simulate and implement a four-channel focus, achieving linear polarization rotation by the finite difference time domain algorithm (FDTD). We adopted the perfectly matched layers (PML) boundary condition for all boundaries to carry out the simulation. The substrate is silicon dioxide, and the thickness is 500 μm. The mark numbers from 1 to 4 and the corresponding focal spots (shown in Figure 3) are located at (5 μm, 5 μm), (−5 μm, 5 μm), (−5 μm, −5 μm), and (5 μm, −5 μm) on the focal plane, respectively. At any diagonal point, the SoPs are orthogonal. According to Equations (2)–(4), when we need to achieve *y*-linearly polarization (YLP) focusing, that is Δφ=π. Similarly, the phase shifts Δφ corresponding to *x*-linearly polarized (XLP) focusing, 45°—linearly polarization focusing, and 135°—linearly polarization focusing are 0, π/2, and 3π/2, respectively. By combining Equations (6)–(10), we can arbitrarily and independently manipulate the wavefronts of a pair of orthogonal CP lights by elaborately designing the parameters of the meta-atoms (Rx,Ry,θ).

The most appropriate set (Rx,Ry,θ) of the meta-atom at the corresponding pixel position can be selected from the meta-atom library (shown in Figure 2a,b by minimizing an error function defined as the maximum error between the required phase and the realized phase profile. Figure 2c illustrates the high polarization conversion efficiencies [38] (with an average value of 90%) at the designed wavelength (λ=1.55 μm), guaranteeing the functionality of the linear polarization rotation meta-device. Based on the strategy of multi-channel phase superposition, we have designed the polarization-rotated metasurface. Figure 2d shows the realized phase profiles conducted by the metasurface operating at the two SOPs, |R> and |L>. These are in agreement with the theoretically required values displayed at the top in Figure 2d. The fine matching between the upper and lower two sets of phase profiles confirms the capability of achieving the multi-channel polarization rotation and focusing with the designed polarization-controlled metasurface. Meta-atoms exhibiting phase differences close to π between *x*- and *y*-polarizations can be selected to meet the condition of half-wave plates, as depicted in Figure 2e.

As depicted in Figure 3a, the focal spots of the four channels on the focal plane show that the polarization angle is evenly spaced at 45 degrees and rotated uniformly from 0–180°. Notably, the polarization states at the diagonal focal points are orthogonal. For instance, 1 and 3 correspond to horizontal and vertical polarizations, while 2 and 4 correspond to 45° and 135° polarizations, respectively. As shown by the blue line in Figure 3b, the overall efficiency of the metalens surpasses 65%, with each channel achieving an efficiency of over 16%, thus confirming the validity of our design method. We also performed polarization analysis in the corresponding orthogonal polarization state of each channel, as shown in the red line in Figure 3b. Each focal spot exhibits a polarization extinction ratio exceeding 100 with a maximum value reaching above 300, ensuring that the intensity signal can be detected reliably.

To verify the target polarization state at each channel, the orthogonal SOP corresponding to each desired focusing channel has been used for polarization analysis. For example, using a 45°—linearly polarization to perform polarization detection on a 135°—linearly polarization at position 2, the normalized intensity distribution shows that the focus at position 2 is almost eliminated. Figure 3d further characterizes the intensity profiles of the horizontal cuts (the red and blue dashed lines in Figure 3c). From the comparison of the normalized intensities, it can be obtained that the polarization states carried by the focusing at different positions are target linear polarization rotation.

### 2.3. Multi-Channel Full-Polarization Generation and Focusing

**Figure 4:**
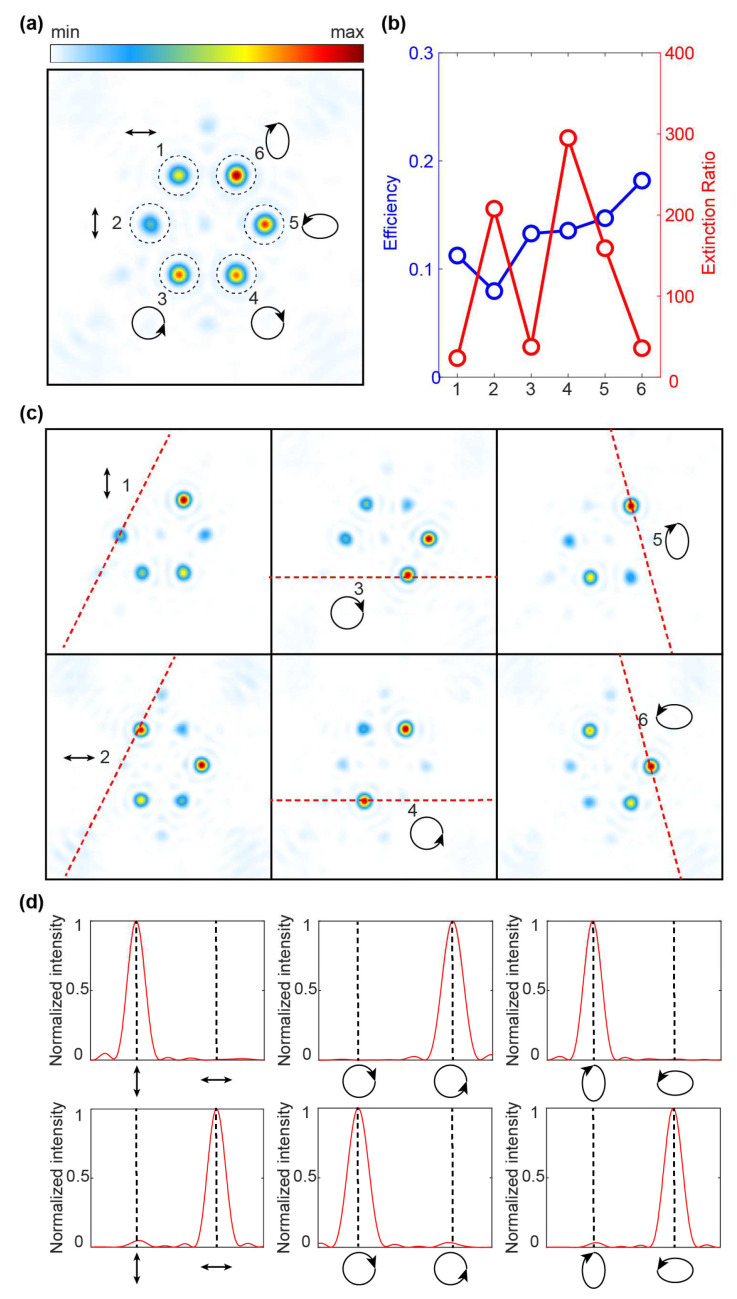
High-efficiency rotated focusing with full-polarization controlled metalens. (**a**) Focal plane intensity distribution with multi-channel focused polarization rotation. Focal spots numbered 1–6 correspond to XLP, YLP, LCP, RCP, EP1, and EP2, respectively. (**b**) Multi-channel focusing efficiency and extinction ratio for different polarization states. (**c**) The intensity distribution of each focus is analyzed using orthogonal SOP. (**d**) The normalized intensity curve corresponding to the focus for each different polarization state is shown as the red dotted line in (**c**).

To further demonstrate the general applicability of the design methodology across various SOPs, we fabricated a six-channel full-polarization focusing metalens. The mark number from 1 to 6 and the corresponding focal spots are located at ($-5\sqrt{2}/2$ μm, $5\sqrt{6}/2$ μm), ($-5\sqrt{2}$ μm, 0), ($-5\sqrt{2}/2$ μm, $-5\sqrt{6}/2$ μm), ($5\sqrt{2}/2$ μm, $-5\sqrt{6}/2$ μm), ($5\sqrt{2}$ μm, 0), and ($5\sqrt{2}/2$ μm, $5\sqrt{6}/2$ μm) on the focal plane, respectively. The corresponding SOP at each position is: ${\left[0,1\right]}^{T}$, ${\left[1,0\right]}^{T}$, ${\left[1,-i\right]}^{T}$, ${\left[1,i\right]}^{T}$, ${\left[1,-3i\right]}^{T}$, and ${\left[3,i\right]}^{T}$, respectively. The polarization state of each channel’s light beam can be expressed using Equation (2). For the case of $A_{R}$ = $A_{L}$ = 1, the output SOP is linearly polarized (LP) light. For a pair of orthogonal CP lights, pure LCP (RCP) corresponds to $A_{R}$ = 0, $A_{L}$ = 2 ($A_{R}$ = 2, $A_{L}$ = 0). We illustrate the output SOP in an arbitrary elliptical polarized (EP) state here with the example of $A_{R}$ = 1, $A_{L}$ = 2 ($A_{R}$ = 2, $A_{L}$ = 1). By selecting different RCP and LCP components illustrated by Equations (3) and (4), we can achieve the generation of an arbitrary SOP. As shown in Figure 4a, for the polarization focusing of the channels numbered 1–6, the final phase shift endowed to LCP and RCP can be expressed as

(11) $$\Phi_{R}=arg\underset{1}{\overset{6}{\sum }}A\cdot \left[e^{i\varphi_{1}^{R}}+e^{i\varphi_{2}^{R}}+2e^{i\varphi_{3}^{R}}+2e^{i\varphi_{5}^{R}}+e^{i\varphi_{6}^{R}}\right]$$

(12) $$\Phi_{L}=arg\underset{1}{\overset{6}{\sum }}A\cdot \left[e^{i\varphi_{1}^{L}}+e^{i{(\varphi }_{2}^{L}+\pi )}+2e^{i\varphi_{4}^{L}}+e^{i\varphi_{5}^{L}}+2e^{i{(\varphi }_{6}^{L}-\pi )}\right]$$

where $A=\sqrt{1/7}$ is the normalized intensity. Following up with the above proposed method, we can independently construct the phase shifts for orthogonal CP incidences via elaborately designing the structural parameters of the meta-atoms based on Equations (6)–(10).

In Figure 4b, the blue line represents an overall efficiency exceeding 75% with the focusing efficiency of each focal spot averaging over 12%. Each channel achieves a minimum polarization extinction ratio above 20 with the maximum exceeding 300. To verify that the polarization state carried by each focal spot on the focal plane is the target design SOP, the corresponding orthogonal polarization is used for polarization analysis, as shown in Figure 4c. For example, using RCP to detect the actual polarization state at position 3, it can be seen from its normalized intensity distribution that the SOP carried by the focal spot at the detection position is LCP. The same applies to the other positions. The normalized intensity cross-sections further characterizing each pair of orthogonal polarizations are shown in Figure 4d. Our results strongly demonstrate that the SOP of each channel satisfies the full-polarization design. The proposed full-polarization generation and multi-channel polarization rotation method is applicable to arbitrary polarization states.

## 3. Conclusions

In summary, we demonstrated a multi-channel interference and methodology for achieving multifunctional meta-devices with arbitrary polarization rotation and focusing. Through effective control of the geometric phase and transmission phase separately, the all-dielectric metasurface realizes multi-channel full-polarization vector control. The multi-channel focusing efficiency up to 75% and the maximum polarization extinction ratio of 300 further validate our interference and spatial-multiplexing methods for controlling arbitrary polarizations. We believe that the design methodology proposed in this article can provide solid and general design strategy for on-chip optical manipulation.

## Data Availability

The data presented in this study are available from the corresponding author upon reasonable request.
